# The dual role of candida glabrata drug:H+ antiporter CgAqr1 (ORF *CAGL0J09944g*) in antifungal drug and acetic acid resistance

**DOI:** 10.3389/fmicb.2013.00170

**Published:** 2013-06-26

**Authors:** Catarina Costa, André Henriques, Carla Pires, Joana Nunes, Michiyo Ohno, Hiroji Chibana, Isabel Sá-Correia, Miguel C. Teixeira

**Affiliations:** ^1^Department of Bioengineering, Instituto Superior Técnico, Technical University of LisbonLisbon, Portugal; ^2^Biological Sciences Research Group, Institute for Biotechnology and Bioengineering, Centre for Biological and Chemical Engineering, Instituto Superior Técnico, Technical University of LisbonLisbon, Portugal; ^3^Medical Mycology Research Center, Chiba UniversityChiba, Japan

**Keywords:** *Candida glabrata*, multidrug resistance, drug:H^+^ antiporters, acetic acid, flucytosine

## Abstract

Opportunistic *Candida* species often have to cope with inhibitory concentrations of acetic acid, in the acidic environment of the vaginal mucosa. Given that the ability of these yeast species to tolerate stress induced by weak acids and antifungal drugs appears to be a key factor in their persistence and virulence, it is crucial to understand the underlying mechanisms. In this study, the drug:H^+^ antiporter CgAqr1 (ORF *CAGL0J09944g*), from *Candida glabrata*, was identified as a determinant of resistance to acetic acid, and also to the antifungal agents flucytosine and, less significantly, clotrimazole. These antifungals were found to act synergistically with acetic acid against this pathogen. The action of CgAqr1 in this phenomenon was analyzed. Using a green fluorescent protein fusion, CgAqr1 was found to localize to the plasma membrane and to membrane vesicles when expressed in *C. glabrata* or, heterologously, in *Saccharomyces cerevisiae*. Given its ability to complement the susceptibility phenotype of its *S. cerevisiae* homolog, ScAqr1, CgAqr1 was proposed to play a similar role in mediating the extrusion of chemical compounds. Significantly, the expression of this gene was found to reduce the intracellular accumulation of ^3^H-flucytosine and, to a moderate extent, of ^3^H-clotrimazole, consistent with a direct role in antifungal drug efflux. Interestingly, no effect of *CgAQR1* deletion could be found on the intracellular accumulation of ^14^C-acetic acid, suggesting that its role in acetic acid resistance may be indirect, presumably through the transport of a still unidentified physiological substrate. Although neither of the tested chemicals induces changes in *CgAQR1* expression, pre-exposure to flucytosine or clotrimazole was found to make *C. glabrata* cells more sensitive to acetic acid stress. Results from this study show that CgAqr1 is an antifungal drug resistance determinant and raise the hypothesis that it may play a role in *C. glabrata* persistent colonization and multidrug resistance.

## INTRODUCTION

Infections caused by *Candida* species are a problem of increasing clinical significance. *Candida glabrata* infections rank second in frequency, immediately after those caused by *C. albicans* (Jarvis, 1995). Although many *Candida* species can be found in the gastrointestinal and genital tract of healthy individuals as innocuous commensals, in immunocompromised hosts they are able to cause skin infections, which may in term lead to invasive infections. In any case, to sense and adapt to different niches within the host environment is essential for their survival and persistence, both as commensals and infection agents.

One of the factors that vary the most within *Candida* colonization sites is pH. Indeed, *Candida* species appear to be suited to thrive in a pH range varying from more than 7.0, as found in the bloodstream, to nearly 4.0, exhibited by the vulvovaginal mucosa. Variation in host niche pH has been seen to affect drug resistance, but the underlying molecular mechanisms are still unclear. Some clues on the mechanisms of the response to pH changes were identified in *C. albicans*. For example, in *C. albicans*, the Rim101 pathway has been shown to be activated under alkaline pH, such as that met during systemic candidiasis (pH 7.4). Rim101 activates the transcription of the *PHR1* gene and represses that of the *PHR2* gene, encoding homologous cell wall β-glycosidases. While Phr1 is required for virulence at alkaline pH, Phr2 is required for virulence at acidic pH, such as that met during vaginal infection (pH 4.0; reviewed in Davis, 2009; Selvig and Alspaugh, 2011). However, little is known on the mechanisms of response and resistance to acidification caused by, or occurring in the presence of weak organic acids, whose concentration reaches quite high values in some colonization or infection sites. Indeed, the concentration of lactic or acetic acid, can reach up to 125 mM in the vaginal tract, particularly under bacterial vaginosis (Chaudry et al., 2004). This is an important issue since the inhibitory effect exerted by weak acids, that dissociate directly in the cytosol leading to intracellular acidification, is much stronger than that exerted by low extracellular pH *per se* (Mira et al., 2010). Furthermore, the presence of inhibitory or close-to-inhibitory concentrations of these weak acids is likely to interfere with the action of antifungal therapy and, presumably, could also play a role in the induction of multidrug resistance acquisition. For example, fluconazole has been shown to act synergistically with acetic acid in its antifungal action against *C. albicans*. Indeed, its fungicidal activity in vaginal fungal infections appears to be due specifically to the natural existence of weak organic acids in this niche (Moosa et al., 2004).

Within the context of factors conferring resistance to weak acids and drugs, it is interesting to point out that some of the multidrug resistance efflux pumps from the major facilitator superfamily (MFS) characterized in *S. cerevisiae* play a role in weak acid stress resistance (Sá-Correia et al., 2009; Mira et al., 2010). This is the case of the drug:H^+^ antiporters Aqr1 (Tenreiro et al., 2002), Azr1 (Tenreiro et al., 2000), Tpo2, and Tpo3 (Fernandes et al., 2005), which have been shown to confer resistance to short chain monocarboxylic acids such as acetic and propionic acids. Among these, Aqr1 stands out as conferring resistance to weak acids, but also to chemical stress inducers such as the antimalarial/antiarrhythmic drug quinidine, the cationic dye crystal violet, or, less clearly, the antifungal drug ketoconazole (Tenreiro et al., 2002). ScAqr1 was further seen to be involved in the excretion of amino acids, particularly homoserine, threonine, alanine, aspartate, and glutamate (Velasco et al., 2004).

This paper describes the functional analysis of the *C. glabrata* *CgAQR1* gene (ORF *CAGL0J09944g*), sharing a high degree of homology with *S. cerevisiae AQR1* gene, with emphasis on its dual role in acetic acid and antifungal drug resistance. The possible synergy of acetic acid and the antifungal drugs to which CgAqr1 confers resistance to and the ability of acetic acid to induce cross-resistance against antifungal drugs was examined. The sub-cellular localization of this transporter was assessed in *C. glabrata* and its action in reducing the intracellular accumulation of ^3^H-flucytosine, ^3^H-clotrimazole, and ^14^C-acetic acid, in *C. glabrata* cells was evaluated. This study provides an insight into the cross-talk between weak acid and antifungal drug action and resistance, as mediated by CgAqr1, with expected impact in the persistence and multidrug resistance phenotypes exhibited by *C. glabrata* within acidic infection sites in the human host.

## MATERIALS AND METHODS

### STRAINS, PLASMIDS, AND GROWTH MEDIA

*Saccharomyces cerevisiae* strain BY4741 (*MAT*a, ura3Δ0, leu2Δ0, his3Δ1, met15Δ0) and the derived single deletion mutant BY4741_Δ*aqr1* were obtained from the Euroscarf collection. The CBS138 *C. glabrata* strain, whose genome sequence was released in 2004, and KUE100 (Ueno et al., 2007) were used in this study. *C. glabrata* strain L5U1 (cgura3Δ0, cgleu2Δ0) was kindly provided by John Bennett (Chen et al., 2007), from the National Institute of Allergy and Infectious Diseases, NIH, Bethesda, USA. The plasmid pGREG576 was obtained from the Drag&Drop collection (Jansen et al., 2005).

Cells were batch-cultured at 30°C, with orbital agitation (250 rpm) in yeast extract peptone dextrose (YPD) growth media, with the following composition: 20 g glucose (Merck), 20 g yeast extract (Difco), and 10 g peptone (Difco). For some of the experiments, minimal medium was used, resulting from different amino acid supplementation of the basal medium (BM) with the following composition (per liter): 1.7 g yeast nitrogen base without amino acids or ${NH}_{4}^{+}$ (Difco), 20 g glucose (Merck), and 2.65 g (NH_4_)_2_SO4 (Merck). *S. cerevisiae* wild-type and derived BY4741 strains were grown in MM4 medium that resulted from BM supplementation with 20 mg/1 methionine, 20 mg/1 histidine, 60 mg/1 leucine, and 20 mg/1 uracil (all from Sigma). *C. glabrata* strains derived from CBS138 and KUE100 or L5U1 were cultured in BM medium without supplementation or with the supplementation of 20 mg/l uracil and 60 mg/l leucine, respectively. To maintain selective pressure over the recombinant strains, the addition of uracil to this medium was only carried out to grow the host yeast cells. Agarized solid media contained, besides the above-indicated ingredients, 20 g/l agar (Iberagar).

### CLONING OF THE *C. glabrata* CgAQR1 GENE (ORF* CAGL0J09944g)*

The pGREG576 plasmid from the Drag&Drop collection (Jansen et al., 2005) was used to clone and express the *C. glabrata* ORF *CAGL0J09944g* in *S. cerevisiae*, as described before for other heterologous genes (Cabrito et al., 2009). pGREG576 was acquired from Euroscarf and contains a galactose inducible promoter (*GAL1*), the yeast selectable marker *URA3* and the *GFP* gene, encoding a green fluorescent protein (GFPS65T), which allows monitoring of the expression and sub-cellular localization of the cloned fusion protein. *CAGL0J09944g* DNA was generated by PCR, using genomic DNA extracted from the sequenced CBS138 *C. glabrata* strain, and the following specific primers: 5′-GAATTCGATATCAAGCTTATCGATACCGTCGACA*ATGGTGGA*AAGTGGTCCAC-3′ and 5′-GCGTGACATAACTAATTACATGACTCGAGGTCGAC*CGTTATCATACTTTTTCTTCAG*-3′. The designed primers contain, besides a region with homology to the first 19 and the last 22 nucleotides of the *CAGL0J09944g* coding region (italic), nucleotide sequences with homology to the cloning site flanking regions of the pGREG576 vector (underlined). The amplified fragment was co-transformed into the parental *S. cerevisiae* strain BY4741 with the pGREG576 vector, previously cut with the restriction enzyme *Sal*I, to obtain the pGREG576_*CgAQR1* plasmid. Since the *GAL1* promoter only allows a slight expression of downstream genes in *C. glabrata*, to visualize by fluorescence microscopy the sub-cellular localization of the *CgAQR1* gene in *C. glabrata*, a new construct was obtained. The *GAL1* promoter present in the pGREG576_*CgAQR1* plasmid was replaced by the copper-inducible *MTI* *C. glabrata* promoter (Willins et al., 2002), giving rise to the pGREG576_MTI_*CgAQR1* plasmid. The *MTI* promoter DNA was generated by PCR, using genomic DNA extracted from the sequenced CBS138 *C. glabrata* strain, and the following specific primers: 5′-TTAACCCTCACTAAAGGGAACAAAAGCTGGAGCTC*TGTACG*ACACGCATCATGTGGCAATC-3′ and 5′-GAAAAGTTCTTCTCCTTTACTCATACTAGTGCGGC*TGTGTTTGTTTTTGTATGTGTT*TGTTG-3′. The designed primers contain, besides a region with homology to the first and last 19 nucleotides of the first 1000 bp of the *MTI* promoter region (italic), nucleotide sequences with homology to the cloning site flanking regions of the pGREG576 vector (underlined). The amplified fragment was co-transformed into the parental strain BY4741 with the pGREG576_*CgAQR1* plasmid, previously cut with *Sac*I and *Not*I restriction enzymes to remove the *GAL1* promoter, to generate the pGREG576_*MTI*_*CgAQR1* plasmid. The recombinant plasmids pGREG576_*CgAQR1* and pGREG576_*MTI*_*CgAQR1* were obtained through homologous recombination in *S. cerevisiae* and verified by DNA sequencing.

### DISRUPTION OF THE *C.glabrata* CgAQR1 GENE (ORF *CAGL0J09944g*)

The deletion of the *CgAQR1* gene was carried out in the parental strain KUE100, using the method described by Ueno et al. (2011). The target gene *CAGL0J09944g* (*CgAQR1*) was replaced by a DNA cassette including the CgHIS3 gene, through homologous recombination. The replacement cassette was prepared by PCR using the following primers: 5′-CGTGATCAGCGGCCCGTTATTATTATAGTTTCTTATCTTTTTTTCGTGATGTCCAAAGTTGCCATGTAAA-3′ and 5′-CCAGCCTCACGATGTGATAACGAAACGAAACTCAAAATTACCCAAAATTACCCACAATCAAAACCTAATA-3′. The pHIS906 plasmid including CgHIS3 was used as a template and transformation was performed as described previously (Ueno et al., 2007). Recombination locus and gene deletion were verified by PCR using the following pair of primers: 5′-CTCGTCGTCAGAGTCGTAGT-3′ and 5′-AGAAAACCAGCCTCACGATG-3′.

### SUSCEPTIBILITY ASSAYS IN *C. glabrata*

The susceptibility of the parental strain KUE100 toward toxic concentrations of the selected drugs and acetic acid was compared to that of the deletion mutant KUE100_*Δcgaqr1* by spot assays or cultivation in liquid growth medium. The ability of *CgAQR1* gene expression to increase wild-type resistance to the tested chemical stresses was also examined through spot assays in the URA3^-^ strain L5U1, using the pGREG576_*CgAQR1* centromeric plasmid.

KUE100 *C. glabrata* cell suspensions used to inoculate the agar plates were mid-exponential cells grown in basal BM medium until culture OD_600__nm_ = 0.4 ± 0.02 was reached and then diluted in sterile water to obtain suspensions with OD_600__nm_ = 0.05 ± 0.005. These cell suspensions and subsequent dilutions (1:5; 1:25) were applied as 4 μl spots onto the surface of agarized BM medium, supplemented with adequate chemical stress concentrations. The tested drugs included the following compounds, used in the specified concentration ranges: the azole antifungal drugs fluconazole (10–200 mg/l), ketoconazole (10–50 mg/l), clotrimazole (1–20 mg/l), tioconazole (0.2–1 mg/l), and miconazole (0.2–1 mg/l), the polyene antifungal drug amphotericin B (0.1–0.5 mg/l), the fluoropyrimidine 5-flucytosine (0.02–5 mg/l), and the antimalarial/antiarrhythmic drug quinidine (3–9 mg/ml; all from Sigma). L5U1 *C. glabrata* cell suspensions used to inoculate the agar plates were mid-exponential cells grown in basal BM medium, containing 0.5% glucose and 0.1% galactose, without uracil when harboring the pGREG576-derived plasmids, until culture OD_600__nm_ = 0.4 ± 0.02 was reached and then diluted in sterile water to obtain suspensions with OD_600__nm_ = 0.05 ± 0.005. These cell suspensions and subsequent dilutions (1:5; 1:25) were applied as 4 μl spots onto the surface of agarized BM medium without uracil, containing 0.1% glucose and 1% galactose, supplemented with adequate chemical stress concentrations, within the above described ranges.

KUE100 *C. glabrata* cell suspensions used for susceptibility assays in liquid medium were grown to mid-exponential phase (OD_600__nm_ = 0.4 ± 0.02) in basal BM medium, harvested by filtration and re-suspended in fresh BM medium, supplemented or not with the specified concentrations of acetic acid, clotrimazole, or 5-flucytosine, with an initial OD_600__nm_ = 0.05 ± 0.005. Growth, taking place in Erlenmeyer flasks, at 37°C, 250 rpm, was followed by measuring the optical density of the cell suspension at 600 nm.

### SUSCEPTIBILITY ASSAYS IN *S. cerevisiae*

The susceptibility of the parental strain BY4741 toward toxic concentrations of the selected drugs and acetic acid was compared to that of the deletion mutant BY4741_*Δaqr1* by spot assays. The ability of *CgAQR1* gene expression to increase wild-type resistance to the tested chemical stresses and to complement the susceptibility phenotype exhibited by the BY4741_*Δaqr1* single deletion mutants was also examined, using the pGREG576_*CgAQR1* centromeric plasmid in which *CgAQR1* is expressed under the *GAL1* promoter.

*Saccharomyces cerevisiae* cell suspensions used to inoculate the agar plates were mid-exponential cells grown in basal MM4-U medium, containing 0.5% glucose and 0.1% galactose, until culture OD_600__nm_ = 0.4 ± 0.02 was reached and then diluted in sterile water to obtain suspensions with OD_600__nm_ = 0.05 ± 0.005. These cell suspensions and subsequent dilutions (1:5; 1:25) were applied as 4 μl spots onto the surface of agarized MM4-U medium, containing 0.1% glucose and 1% galactose, supplemented with adequate chemical stress concentrations. The tested drugs and other xenobiotics included the following compounds, used in the specified concentration ranges: the azole antifungal drugs fluconazole (50–200 mg/l), itraconazole (40–80 mg/l), ketoconazole (10–50 mg/l), clotrimazole (1–20 mg/l), tioconazole (0.05–0.2 mg/l), and miconazole (0.05–0.2 mg/l), the polyene antifungal drug amphotericin B (0.05–0.5 mg/l), the fluoropyrimidine 5-flucytosine (0.02–5 mg/l), and the weak organic monocarboxylic acid acetic acid (30–60 mM) (all from Sigma).

### *CgAqr1* SUB-CELLULAR LOCALIZATION ASSESSMENT

The sub-cellular localization of the CgAqr1 protein was determined based on the observation of BY4741 *S. cerevisiae* or L5U1 *C. glabrata* cells transformed with the pGREG576-*CgAQR1* or pGREG576-*MTI*-*CgAQR1* plasmids, respectively. These cells express the CgAqr1_GFP fusion protein, whose localization may be determined using fluorescence microscopy. *S. cerevisiae* cell suspensions were prepared by cultivation in MM4-U medium, containing 0.5% glucose and 0.1% galactose, at 30°C, with orbital shaking (250 rev/min), until a standard culture OD_600__nm_ (Optical Density at 600 nm) = 0.4 ± 0.04 was reached. At this point cells were transferred to the same medium containing 0.1% glucose and 1% galactose, to induce protein expression. *C. glabrata* cell suspensions were prepared in BM-U medium, until a standard culture OD_600__nm_ = 0.4 ± 0.04 was reached, and transferred to the same medium supplemented with 30 μM CuSO_4_ (Sigma), to induce protein over-expression. After 5 h of incubation, the distribution of CgAqr1_GFP fusion protein in *S. cerevisiae* or in *C. glabrata* living cells was detected by fluorescence microscopy in a Zeiss Axioplan microscope (Carl Zeiss MicroImaging), using excitation and emission wavelength of 395 and 509 nm, respectively. Fluorescence images were captured using a cooled CCD camera (Cool SNAPFX, Roper Scientific Photometrics).

### TRANSPORT ASSAYS USING RADIOLABELED COMPOUNDS

[^3^H]-flucytosine, [^3^H]-clotrimazole, and [^14^C]-acetate accumulation assays were carried out as described before (Vargas et al., 2004). To estimate the accumulation of each radiolabeled compound (intracellular/extracellular) in yeast cells, the parental strain KUE100 and the mutant strain KUE100_*Δcgaqr1* were grown in BM medium till mid-exponential phase and harvested by filtration. Cells were washed and re-suspended in BM medium, to obtain dense cell suspensions [OD_600__nm_ = 5.0 ± 0.1, equivalent to approximately 2.2 mg (dry weight)/ml]. After 5 min incubation at 30°C, with agitation (150 rpm), the radiolabeled compound was added to the cell suspensions [1 μM of [^3^H]-flucytosine (American Radiolabeled Chemicals; 1 mCi/ml) and 3 mg/l of unlabeled flucytosine; or 0.1 μM of [^3^H]-clotrimazole (American Radiolabeled Chemicals; 1 mCi/ml) and 75 mg/l of unlabeled clotrimazole; 3.5 μM of [^14^C]-acetic acid (American Radiolabeled Chemicals; 0.1 mCi/ml) and 100 mM of unlabeled acetic acid]. The accumulation of each radiolabeled compound was followed, in individual assays, for an additional period of 30 min until equilibrium was reached. In all cases, the intracellular accumulation of the radiolabeled compound was followed by filtering 200 μl of cell suspension, at adequate time intervals, through pre-wetted glass microfiber filters (Whatman GF/C). The filters were washed with ice-cold TM buffer and the radioactivity measured in a Beckman LS 5000 TD scintillation counter. Extracellular concentration of the radiolabeled compound was estimated, by radioactivity assessment of 50 μl of the supernatant.

Non-specific adsorption of each radiolabeled compound to the filters and to the cells (less than 5% of the total radioactivity) was assessed and taken into consideration. To calculate the intracellular concentration of each radiolabeled compound, the internal cell volume (*V*_i_) of the exponential cells, grown in the absence of drug and used for accumulation assays, was considered constant and equal to 2.5 μl/mg dry weight (Rosa and Sá-Correia, 1996).

### *CgAqr1* EXPRESSION MEASUREMENTS

The levels of *CgAQR1* transcripts in *C. glabrata* cells were assessed by quantitative real-time PCR. Total RNA samples were obtained from cell suspensions harvested in control conditions (mid-exponential phase cells in the absence of drugs) or upon 1 h of exposure to 75 mg/l clotrimazole or 3.5 mg/l flucytosine or 60 mM acetic acid. Synthesis of cDNA for real-time RT-PCR experiments, from total RNA samples, was performed using the Multiscribe^TM^ reverse transcriptase kit (Applied Biosystems) and the 7500 RT-PCR Thermal Cycler Block (Applied Biosystems), following the manufacturer’s instructions. The quantity of cDNA for the following reactions was kept around 10 ng. The subsequent RT-PCR step was carried out using SYBR^®^ Green reagents. Primers for the amplification of the *CgAQR1* and *CgACT1* cDNA were designed using Primer Express Software (Applied Biosystems) and are 5′-GCTGATAAGTTCGGCCGTAGA-3′ and 5′-AATGGAGGCAACCACGTAGATC-3′ and 5′-AGAGCCGTCTTCCCTTCCAT-3′ and 5′-TTGACCCATACCGACCATGA-3′, respectively. The RT-PCR reaction was carried out using a thermal cycler block (7500 Real-Time PCR System – Applied Biosystems). Default parameters established by the manufacturer were used and fluorescence detected by the instrument and registered in an amplification plot (7500 System SDS Software – Applied Biosystems). The *CgACT1* mRNA level was used as an internal control. The relative values obtained for the wild-type strain in control conditions were set as 1 and the remaining values are presented relative to that control. To avoid false positive signals, the absence of non-specific amplification with the chosen primers was confirmed by the generation of a dissociation curve for each pair of primers.

## RESULTS

### *CgAqr1* EXPRESSION CONFERS RESISTANCE TO ACETIC ACID

**FIGURE 1:**
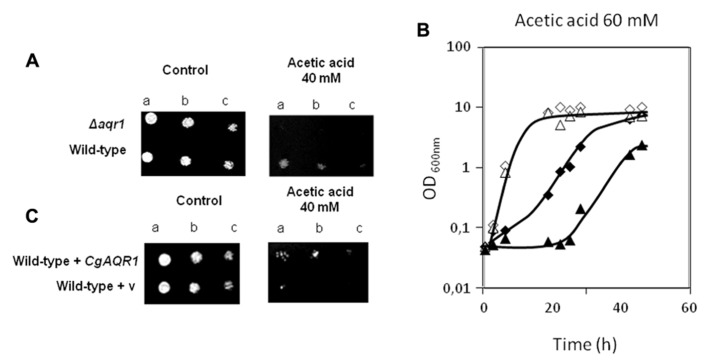
**(A)** Comparison of the susceptibility to inhibitory concentrations of acetic acid, at the indicated concentration, of the *C. glabrata* KUE100 and KUE100_Δ cgaqr1 strains, in BM agar plates by spot assays.**(B)**Comparison of growth curves of the *C. glabrata* KUE100 (◊,◆) and KUE100_*Δcgaqr1* (Δ,▲) strains, in liquid BM medium, supplemented with 0 mM (◊,Δ) or 60 mM (◆,▲) of acetic acid.**(C)**Comparison of the susceptibility to inhibitory concentrations of acetic acid, at the indicated concentration, of the *C. glabrata* L5U1 strain, harboring the pGREG576 cloning vector (v) or the pGREG576_*CgAqr1* plasmid in BM agar plates, without uracil, by spot assays. The inocula were prepared as described in Section “Materials and Methods.” Cell suspensions used to prepare the spots were 1:5 (b) and 1:25 (c) dilutions of the cell suspension used in (a). The displayed images and growth curves are representative of at least three independent experiments.

Given the previously observed effect of *S. cerevisiae AQR1* gene in weak acid stress tolerance (Tenreiro et al., 2002), the action of CgAqr1 in acetic acid tolerance was inspected. The deletion of *CgAQR1* gene in *C. glabrata* clearly decreases its tolerance to an inhibitory concentration of acetic acid (**Figure 1**). This is visible both in spot assays (**Figure 1A**) and in liquid medium cultivation (**Figure 1B**). Sudden exposure of un-adapted wild-type *C. glabrata* cells to 60 mM acetic acid leads to 5 h of lag-phase followed by growth resumption with reduced kinetics, while cells devoid of CgAqr1 enter a lag-phase that lasts for around 18 h, before growth resumption. Apparently, the absence of the Aqr1 gene affects only slightly the growth rate at which cells are able to reassume exponential growth in the presence of acetic acid, suggesting that its role is mainly played during the adaptation to this stress (Table 1). The introduction of a recombinant plasmid expressing *CgAqr1* further increases *C. glabrata* natural resistance toward acetic acid, when compared to the same strain harboring the corresponding cloning vector (**Figure 1C**), reinforcing the finding that *CgAqr1* is a determinant of acetic acid resistance in *C. glabrata*.

### *CgAqr1* EXPRESSION CONFERS RESISTANCE TO FLUCYTOSINE AND AZOLE ANTIFUNGAL DRUGS

**FIGURE 2:**
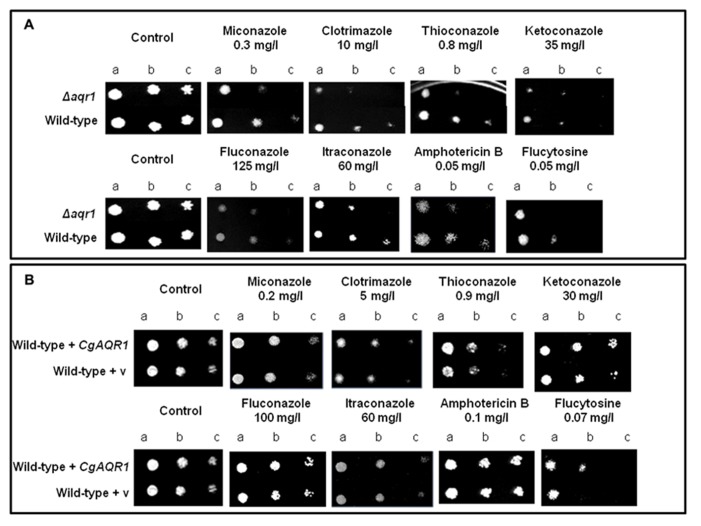
**(A)** Comparison of the susceptibility to antifungal drugs, at the indicated concentrations, of the *C. glabrata *KUE100 and KUE100_*Δcgaqr1* strains, in BM agar plates by spot assays. **(B)** Comparison of the susceptibility to antifungal drugs, at the indicated concentrations, of the *C. glabrata *L5U1 strain, harboring the pGREG576 cloning vector (v) or the pGREG576_*CgAqr1* plasmid in BM agar plates, without uracil, by spot assays. The inocula were prepared as described in Section “Materials and Methods.” Cell suspensions used to prepare the spots were 1:5 (b) and 1:25 (c) dilutions of the cell suspension used in (a). The displayed images are representative of at least three independent experiments.

**FIGURE 3:**
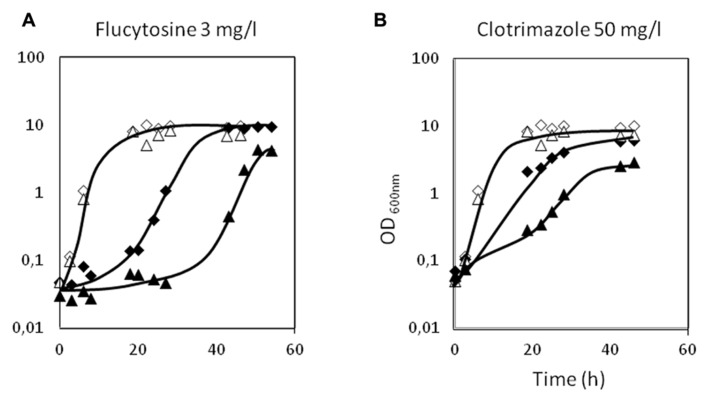
**Comparison of the growth curves of *C. glabrata* KUE100 (◊,◆) and KUE100_Δ*cgaqr1* (Δ,▲) strains, in liquid BM medium, in the absence (◊,Δ) or presence of 3 mg/l flucytosine **(A)** or 50 mg/l clotrimazole **(B)** (◆,▲). The inocula were prepared as described in Section “Materials and Methods.”** The displayed growth curves are representative of at least three independent experiments.

The deletion of the *CgAqr1* gene in *C. glabrata* was found, based on spot assays, to increase the susceptibility of this pathogen against the antifungal fluoropyrimidine analog flucytosine, and the imidazole antifungal drugs miconazole, clotrimazole, and tioconazole (**Figure 2A**). The introduction of a recombinant plasmid expressing *CgAqr1* increases *C. glabrata* natural resistance toward flucytosine, but only very mildly against clotrimazole and tioconazole, when compared to the same strain harboring the corresponding cloning vector (**Figure 2B**), reinforcing the finding that *CgAqr1* is a strong determinant of flucytosine resistance in *C. glabrata*. No effect of CgAqr1 expression could be clearly detected in *C. glabrata* susceptibility to fluconazole, itraconazole, or amphotericin B. The comparison of the growth curves of wild-type and derived *Δcgaqr1* cells in liquid medium show that the deletion of CgAqr1 clearly impairs *C. glabrata* growth in the presence of clotrimazole and, particularly, flucytosine (**Figure 3**). Indeed, although the growth curves of both strains are nearly indistinguishable in control conditions, upon sudden exposure to the presence of 3 mg/ml of flucytosine the wild-type cells experience a period of around 18 h of lag-phase, followed by exponential growth with a reduced rate, while the *Δcgaqr1* deletion mutant cells enter a period of lag-phase that last for around 35 h prior to growth resumption with even more inhibited growth kinetics (Table 1; **Figure 3A**). A less striking, but still clear, effect of CgAqr1 deletion can also be seen in the presence of 50 mg/ml of clotrimazole. *Δcgaqr1* deletion mutant cells exhibit a period of lag-phase of around 18 h prior to growth resumption, while the effect of this concentration of clotrimazole in wild-type cells appears to be felt mostly at the level of growth rate inhibition (Table 1; **Figure 3B**).

**Table 1:** Comparison of the susceptibility to inhibitory concentrations of acetic acid, clotrimazole, or flucytosine of the *C. glabrata *KUE100 and KUE100_*Δcgaqr1* strains, based on the growth parameters lag-phase duration, exponential growth rate, and final biomass.

Growth conditions	Strain	Lag-phase (h)	Growth rate/h	Final biomass (OD_**600**__**nm**_)
Control	Wild-type	nd	1.32 ± 0.08	9.76 ± 0.39
	*Δaqr1*	nd	1.23 ± 0.09	6.64 ± 0.96
Clotrimazole	Wild-type	6.25 ± 2.71	0.20 ± 0.04	6.06 ± 0.09
50 mg/l	*Δaqr1*	17.96 ± 0.27	0.19 ± 0.01	3.00 ± 0.17
Flucytosine	Wild-type	18.76 ± 0.13	0.30 ± 0.02	9.28 ± 0.51
3 mg/l	*Δaqr1*	35.14 ± 3.60	0.21 ± 0.07	4.06 ± 0.37
Acetic acid	Wild-type	5.31 ± 2.86	0.19 ± 0.02	7.76 ± 0.45
60 mM	*Δaqr1*	18.28 ± 1.08	0.14 ± 0.001	2.36 ± 0.01

*Values are the average of at least three independent experiments ± standard deviation. Nd, not detected.*

**FIGURE 4:**
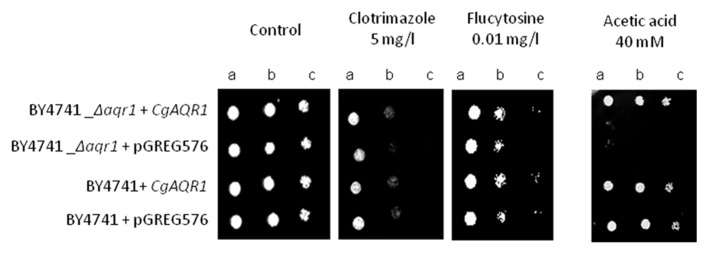
**Comparison of the susceptibility to antifungal drugs and acetic, at the indicated concentrations, of the *S. cerevisiae* BY4741 and BY4741_*Δaqr1* strains, harboring the cloning vector pGREG576 (v) or the derived *cgaqr1* expression plasmid pGREG_*cgaqr1*, in MM4 agar plates by spot assays.** Cell suspensions used to prepare the spots were 1:5 (b) and 1:25 (c) dilutions of the cell suspension used in (a). The inocula were prepared as described in Section “Materials and Methods.” Images are representative of at least three independent experiments.

Using *S. cerevisiae* as a heterologous expression system, the effect of *cgaqr1* expression in yeast resistance to antifungal drugs was further tested, in order to verify whether or not *cgaqr1* is able to functionally complement its *S. cerevisiae* homolog. The deletion of the* S. cerevisiae AQR1* gene was found to increase the susceptibility toward flucytosine, clotrimazole and, as observed before (Tenreiro et al., 2002), acetic acid exhibited by the corresponding parental strain (**Figure 4**). When expressed in the *S. cerevisiae* *Δaqr1* background, the *cgaqr1* gene was able to rescue all the observed susceptibility phenotypes, further confirming its role in flucytosine and imidazole drug resistance (**Figure 4**).

### ACETIC ACID ACTS SYNERGISTICALLY WITH CLOTRIMAZOLE AND FLUCYTOSINE

**FIGURE 5:**
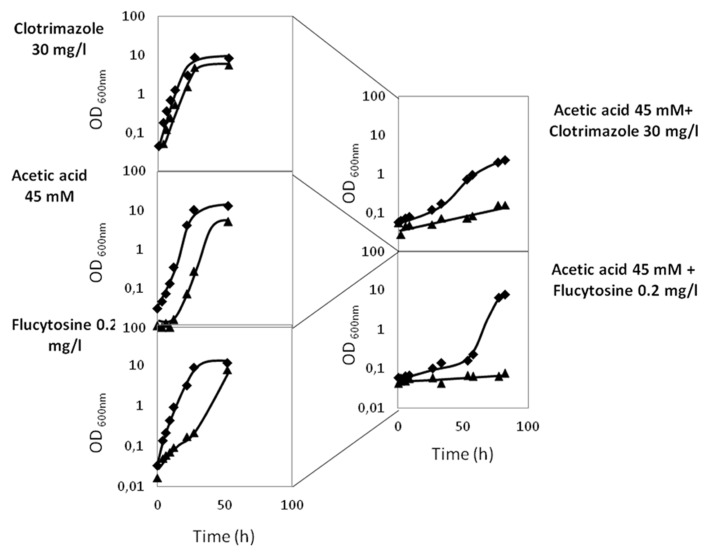
**Comparison of the growth curves of *C. glabrata* KUE100 (◆) and KUE100_*Δcgaqr1* (▲) strains, in liquid BM medium, in the presence of clotrimazole, acetic acid, or flucytosine (on the right) or in the simultaneous presence of acetic acid and clotrimazole or acetic acid and flucytosine (on the left), at the indicated concentrations.** The inocula were prepared as described in Section “Materials and Methods.” The displayed growth curves are representative of at least three independent experiments.

In order to evaluate the eventual effect of acetic acid, present in some of the natural niches of *Candida* infection, in antifungal therapy, the effect of co-exposure of *C. glabrata* cells to acetic acid and to the antifungals clotrimazole and flucytosine was examined. *C. glabrata* wild-type cells were exposed to concentrations of acetic acid, clotrimazole, and flucytosine that, individually, do not affect growth: 45 mM, 30 and 0.2 mg/l, respectively (**Figure 5**). However, when in combination, the same “innocuous” concentrations of acetic acid and clotrimazole or acetic acid and flucytosine, were found to lead to severe growth impairment. Indeed, when grown in the presence of 30 mg/l clotrimazole plus 45 mM of acetic acid, the *C. glabrata* population enters a period of 40 h of lag-phase, followed by a drastically reduced growth rate and reaching lower levels of final biomass when compared to control cells (**Figure 5**). Sudden exposure to 0.2 mg/l flucytosine plus 45 mM of acetic acid leads to an even longer period of lag-phase (50 h), upon which growth resumption is achieved (**Figure 5**). It is clear that the inhibitory effect of *C. glabrata* exposure to acetic acid plus flucytosine or acetic acid plus clotrimazole is much stronger than the sum of their individual inhibitory effects, suggesting that acetic acid acts synergistically with flucytosine and clotrimazole. In the absence of CgAqr1, this effect seems to be even more pronounced. Given the fact that flucytosine is used mostly in combination with fluconazole, we further verified experimentally that flucytosine and fluconazole do exhibit a synergistic effect. However, and as expected based on the fact that CgAqr1 does not confer resistance to fluconazole, no increased role of CgAqr1 in the presence of this combination of drugs was observed (results not shown).

### *CgAqr1* IS LOCALIZED TO THE PLASMA MEMBRANE AND MEMBRANE VESICLES IN *C. glabrata* AND IN *S. cerevisiae*

**FIGURE 6:**
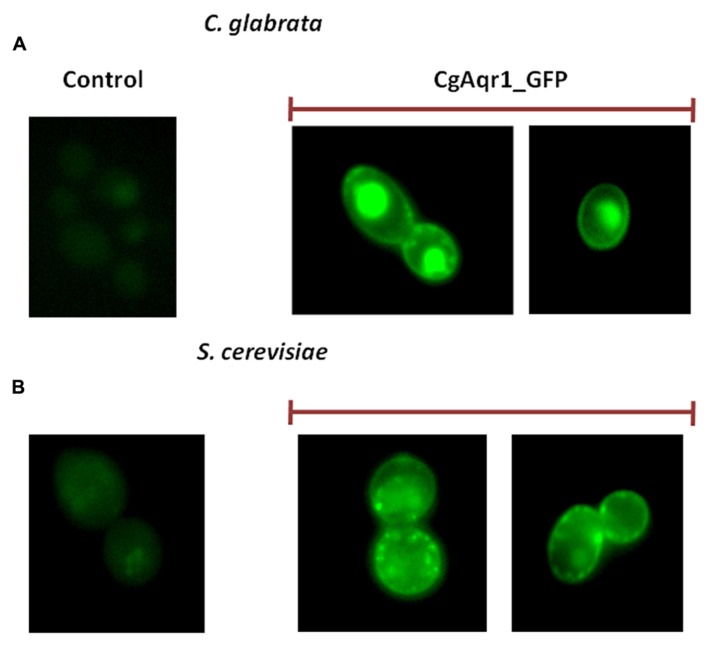
**Fluorescence of exponential phase L5U1 *C. glabrata* cells (A) or BY4741 *S. cerevisiae* cells **(B)**, harboring the cloning vector pGREG576 (control) or the pGREG576_*CgAqr1* or pGREG576_ MTI_*CgAqr1* plasmids (CgAqr1_GFP), after 5 h of galactose- or copper-induced recombinant protein production, respectively.** Results indicate that the CgAqr1-GFP fusion protein localizes to the plasma membrane and membrane vesicles in both *S. cerevisiae* and *C. glabrata* cells.

*Candida glabrata* cells harboring the pGREG576_*MTI*_*CgAQR1* plasmid were grown to mid-exponential phase in minimal medium, and then transferred to the same medium containing 30 μM CuSO_4_, to promote protein expression in moderate controlled levels. At a standard OD_600__nm_ of 0.5 ± 0.05, obtained after around 5 h of incubation, cells were inspected through fluorescence microscopy. This period of incubation was found to allow detectable protein expression levels, but not a high degree of over-expression that may lead to mis-localization. In *C. glabrata* cells, the CgAqr1_GFP fusion was found to be localized to the cell periphery, and also to a punctuate distribution throughout the cell (**Figure 1A**). Control cells, on the other hand, harboring the pGREG576 cloning vector, displayed a slight and uniform distribution of fluorescence (**Figure 6A**), similar to what can be observed as the host cells auto-fluorescence. Since CgAqr1 is predicted to be an integral membrane protein (Gbelska et al., 2006), these results strongly suggest a plasma membrane and, eventually, membrane vesicle localization, similar to what was observed for its *S. cerevisiae* homolog Aqr1 (Tenreiro et al., 2002; Velasco et al., 2004). *S. cerevisiae* cells harboring the pGREG576_*CgAqr1* plasmid were also tested for the sub-cellular localization of CgAqr1, to verify that in these cells, the *C. glabrata* transporter was similarly localized to the plasma membrane and membrane vesicles. Cells were grown to mid-exponential phase in minimal medium containing 0.5% glucose and 0.1% galactose, and then transferred to the same medium containing 0.1% glucose and 1% galactose, to promote protein over-expression. At a standard OD_600__nm_ of 0.5 ± 0.05, obtained after around 5 h of incubation, cells were inspected through fluorescence microscopy and plasma membrane and membrane vesicle localization was verified (**Figure 6B**).

### *CgAqr1* PLAYS A ROLE IN REDUCING THE INTRACELLULAR ACCUMULATION OF ^3^H-flucytosine, BUT NOT OF ^3^H-CLOTRIMAZOLE OR ^14^C-ACETIC ACID IN *C. glabrata*

**FIGURE 7:**
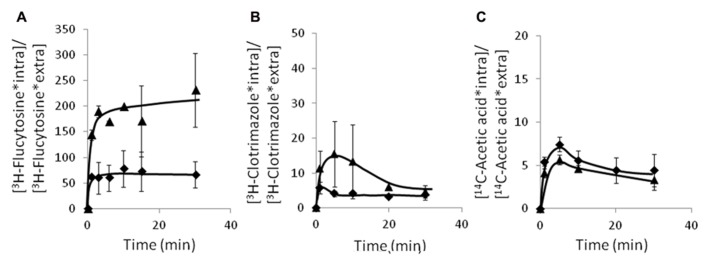
**Time course accumulation ratio of [^3^H]-flucytosine **(A)**, [^**3**^H]-clotrimazole **(B)**, and [^**14**^C]-acetate **(C)** in non-adapted cells of KUE100 (◆) or KUE100_*Δcgaqr1* (▲) strains, during cultivation in BM liquid medium in the presence of 3 mg/l, 30 mg/l, or 100 mM of unlabeled flucytosine, clotrimazole, or acetic acid, respectively.** The accumulation ratio values are averages of at least three independent experiments. Error bars represent the corresponding standard deviations.

Since the *C. glabrata* gene *CgAqr1*, was found herein to encode a drug resistance transporter of the plasma membrane, and of what appears to be the membrane of exocytic vesicles, and to act as a determinant of resistance to flucytosine, acetic acid, and less significantly, clotrimazole, its possible involvement in reducing the accumulation of these compounds in challenged yeast cells was examined. The accumulation of radiolabeled flucytosine was seen to be four times higher in cells devoid of CgAqr1 than in wild-type cells (**Figure 7A**), correlating with the strong effect of *CgAQR1* deletion in flucytosine resistance (**Figure 3**). The accumulation of [^3^H]-labeled clotrimazole in non-adapted *C. glabrata* cells suddenly exposed to the presence of 30 mg/l cold clotrimazole was also tested and found to be slightly higher than in cells devoid of CgAqr1 than in parental KUE100 cells (**Figure 7B**), which appears to be consistent with the relatively small difference in growth inhibition exhibited by wild-type and *Δcgaqr1* cells. Surprisingly, no clear difference in ^14^C-acetic acid accumulation could be found in wild-type and *Δcgaqr1* strains (**Figure 7C**). These results strongly suggests that CgAqr1 activity increases yeast resistance toward flucytosine by reducing its accumulation within yeast cells, presumably by catalyzing the direct extrusion of this antifungal drug, while its action in acetic acid stress tolerance may be indirect.

### *CgAqr1* TRANSCRIPT LEVELS ARE NOT UP-REGULATED UNDER ACETIC ACID, CLOTRIMAZOLE, OR FLUCYTOSINE STRESS

**FIGURE 8:**
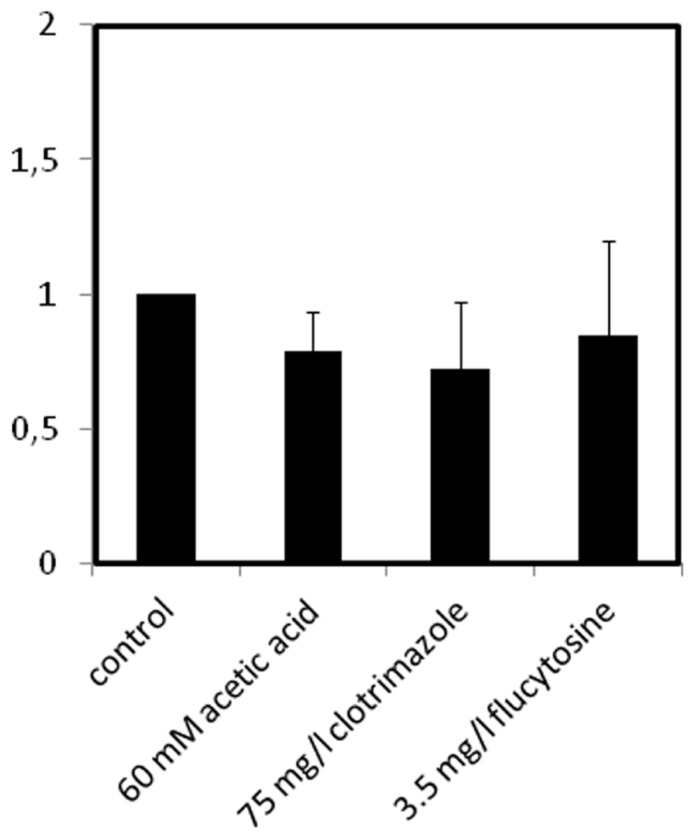
**Comparison of the variation of the *CgAQR1* transcript levels in wild-type *C. glabrata* cells before (control) and after 1 h of exposure to acetic acid-, clotrimazole-, or flucytosine-induced stress, at the indicated concentrations.** The presented transcript levels were obtained by quantitative RT-PCR, as described in Section “Materials and Methods,” and are relative *CgAQR1/CgACT1* mRNA values, considering the value registered in control conditions, equal to 1. The obtained values are the average of at least three independent experiments. Error bars represent the corresponding standard deviations.

The effect of *C. glabrata* cell exposure to inhibitory concentrations of acetic acid, clotrimazole or flucytosine, to which CgAqr1 confers resistance, in *CgAqr1* transcription was evaluated. The transcript levels of *CgAqr1* gene were seen to suffer no significant change upon 1 h of exposure of an un-adapted *C. glabrata* population to inhibitory concentrations of acetic acid, clotrimazole or flucytosine (**Figure 8**).

### ACETIC ACID DOES NOT PROVIDE CROSS-PROTECTION AGAINST CLOTRIMAZOLE AND FLUCYTOSINE

**FIGURE 9:**
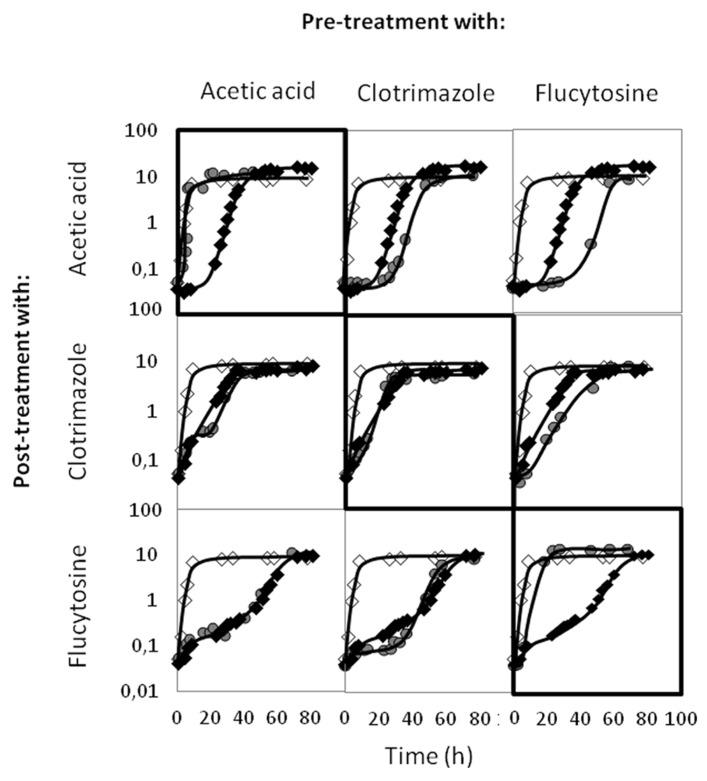
**Comparison of the growth curves of *C. glabrata *KUE100 strain, in liquid BM medium, in the absence (◊) or presence of acetic acid, clotrimazole, or flucytosine (◆,** 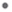), **as indicated on the left.** The inocula were prepared as described in Section “Materials and Methods,” from exponentially growing cells that were growing in control conditions (◆), or that were pre-adapted (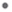) to the stress inducer indicated on the top. The displayed growth curves are representative of at least three independent experiments.

Given that CgAqr1 provides *C. glabrata* protection against acetic acid, flucytosine, and clotrimazole, the possibility that exposure to each of these stress agents might confer cross-protection against the remaining was hypothesized. To evaluate this possibility, *C. glabrata* cells were pre-exposed to 60 mM of acetic acid, 50 mg/l of clotrimazole, and 0.5 mg/l of flucytosine and cultivated until mid-exponential phase was reached (OD_600__nm_ = 1.5 ± 0.05). These cells, growing exponentially in the presence of each of these stress agents were then harvested, washed, and exposed to the three growth inhibitors as secondary stress agents. Pre-exposure to acetic acid made *C. glabrata* cells able to growth at maximal exponential rate upon re-exposure to the same concentration of the acid, but did not increase yeast resistance to clotrimazole or flucytosine (**Figure 9**). On the other hand, pre-exposure to clotrimazole appears to have no protective effect against flucytosine or, not even, to re-exposure to clotrimazole itself, and to induce a sensitization against post-exposure to acetic acid (**Figure 9**). Finally, pre-exposure to flucytosine, while making *C. glabrata* cells tolerant to re-exposure to flucytosine did actually make the cells more susceptible to acetic acid and clotrimazole (**Figure 9**).

## DISCUSSION

In this study, the functional characterization of the *C. glabrata* CgAqr1 drug:H^+^ antiporter was carried out. CgAqr1 was identified as the first of its family to confer resistance in this species to the antifungal drug flucytosine, but also to acetic acid.

To the best of our knowledge, no multidrug resistance transporter had ever been linked to flucytosine resistance. This is quite surprising, considering that it has been known for some time that the acquisition of flucytosine resistance among clinical isolates occurs very fast, which appears to be compatible with the action of drug efflux pumps (Thompson et al., 2009). In most cases, flucytosine resistance has been associated to point mutations or changes in the expression of the genes involved in the uptake (e.g., carried out by cytosine permeases, such as Fcy2) and catabolism (the first step carried out by cytosine deaminase Fcy1) of this antifungal pro-drug (Hope et al., 2004). Significantly, results from this study point out to a direct role of CgAqr1 in flucytosine efflux. Indeed, the increased flucytosine resistance observed in cells expressing the *CgAQR1* gene, when compared to *Δcgaqr1* deletion mutant cells, appears to correlate with the observation that the accumulation of this antifungal drug is twofold higher in the deletion mutant, when compared to the corresponding parental strain. The results displayed herein further highlight the unexplored role of drug efflux pumps in the context of flucytosine resistance.

CgAqr1 was also found to confer, to a lower level, resistance to azole drugs, including the imidazoles miconazole, tioconazole, and clotrimazole, used in the treatment of fungal skin infections and vaginal or oral candidemia. In *C. glabrata*, resistance to azole drugs in clinical isolates has been shown to depend often on the action of the ABC drug efflux pumps encoded by *CgCDR1*, *CgCDR2*, and *CgAUS1* (Cannon et al., 2009). Very recently, the *C. glabrata* CgQdr2 drug:H^+^ antiporter was identified as the first of its family to confer imidazole drug resistance, playing a direct role in the extrusion of clotrimazole (Costa et al., 2013). In this study, CgAqr1 is added to the number of characterized *Candida* transporters involved in azole drug resistance. However, the deletion of *CgAQR1* was found to have only a slight effect on ^3^H-clotrimazole accumulation in *C. glabrata* cells, consistent with the moderate increase in susceptibility registered upon *CgAQR1* deletion.

Based on its high degree of homology and functional similarity to the *S. cerevisiae AQR1* gene, a possible physiological role for CgAqr1 linked to yeast survival in the presence of inhibitory concentrations of acetic acid (Tenreiro et al., 2002) was also inspected. Indeed, CgAqr1 expression did improve *C. glabrata* and *S. cerevisiae* fitness under inhibitory concentrations of this weak carboxylic acid, and complemented the *S. cerevisiae* *Δaqr1* susceptibility phenotype observed in these conditions. ScAqr1 and CgAqr1 were further found to confer flucytosine resistance in *S. cerevisiae*, CgAqr1 being able to complement the absence of its *S. cerevisiae* counterpart under flucytosine stress. The notion that ScAqr1 and CgAqr1 may have overlapping functions is reinforced by the finding that their sub-cellular localization is also quite similar, while different from that of the remaining members of their family (Sá-Correia et al., 2009). Indeed, similar to what had been registered for ScAqr1 (Tenreiro et al., 2002; Velasco et al., 2004), CgAqr1 was found to be localized to both the plasma membrane and membrane vesicles, being proposed to catalyze the extrusion of its substrates across the plasma membrane or through exocytic vesicles. The possibility that CgAqr1 displays as physiological role the catalysis of acetate excretion was further investigated, but no effect of *CgAQR1* expression on ^14^C-acetate accumulation could be detected. Significantly, the expression of the *S. cerevisiae* Aqr1 transporter had also been found to have no effect on acetate transport (Tenreiro et al., 2002). Although the exact role of CgAqr1 in acetic acid resistance remains to be perceived, it may be indirectly due to the transport of a still unidentified physiological substrate. Since ScAqr1 was seen to be involved in the excretion of amino acids, particularly homoserine, threonine, alanine, aspartate, and glutamate (Velasco et al., 2004), it is reasonable to hypothesize that a similar trait may be exhibited by its ortholog in *C. glabrata*. Although it appears difficult to foresee how exactly amino acid excretion may contribute to acetic acid and azole drug resistance, the truth is that the molecular mechanisms behind the apparent promiscuity exhibited by MDR transporters of the ABC and MFS superfamilies remains elusive and controversial (Roepe et al., 1996; Prasad et al., 2002; Sá-Correia and Tenreiro, 2002; Paulsen, 2003; Jungwirth and Kuchler, 2006; Sá-Correia et al., 2009). Furthermore, the fact that some of the compounds to which multidrug transporters confer resistance to are not extruded through their direct action has been observed for the majority of the ABC and MFS–MDR transporters characterized in *S. cerevisiae* (Jungwirth and Kuchler, 2006; Sá-Correia et al., 2009), whereas they have been found to be involved in the transport of physiological substrates, including membrane lipids and ions that in turn affect the plasma membrane partition, permeability or toxicity of drugs, and xenobiotics (Vargas et al., 2007; Cabrito et al., 2011; Teixeira et al., 2011, 2012). Such a possible scenario can also be envisaged to explain how CgAqr1 may confer acetic acid resistance, without affecting its intracellular accumulation.

Tolerance against weak acids is an important feature for *Candida* species to thrive in the acidic environment (pH ~ 4) of the vaginal tract, where significant concentrations of acetic and lactic acids can be found (Davis, 2009). Furthermore, *C. glabrata* is able to survive inside the phagolysosome, where it has to deal with hydrolytic enzymes and an acidic environment. Results from this study point out to the existence of a synergistic action between both flucytosine and clotrimazole and acetic acid, a phenomenon observed previously between acetic acid and fluconazole in *C. albicans* (Moosa et al., 2004), reinforcing the importance of weak acid resistance mechanisms in the context of antifungal therapy. This effect, coupled with the dual role of CgAqr1 in acetic acid and antifungal drug resistance led us to hypothesize that pre-exposure of *C. glabrata* cells to acetic acid concentrations similar to those found in the vaginal tract might contribute to make them more tolerant to antifungal drugs. However, the predicted cross-resistance effect was not observed. Indeed, pre-exposure to acetic acid had no effect on the susceptibility of *C. glabrata* cells to flucytosine or clotrimazole, whereas pre-exposure to clotrimazole and flucytosine sensitizes these cells toward acetic acid stress. Interestingly, *CgAqr1* transcript levels in *C. glabrata* were seen to be irresponsive to chemical stress exposure, either that induced by acetic acid, flucytosine, or clotrimazole, suggesting that other genes or post-translation regulatory effects may underlie the antifungal drug-induced sensitization of *C. glabrata* cells. The fact that CgAqr1 confers resistance to both antifungal drugs and acetic acid strongly suggests that this transporter may play an important role in the persistence of *C. glabrata* infections in acidic loci.

Interestingly, the deletion of *CgAQR1* was found to play a major role in decreasing stress-induced lag-phase duration, while having only a moderate effect in *C. glabrata* maximal growth rate in the presence of flucytosine, acetic acid, or clotrimazole. A similar phenomenon was observed for most of the CgAqr1 homologs in *S. cerevisiae* (Sá-Correia et al., 2009). The current model to explain this phenotype proposes that drug efflux pumps are mostly required to deal with sudden exposure to chemical stress. Additional mechanisms leading to the impermeabilization of the cell envelope are reported to be activated upon chemical stress exposure, preventing drug re-entrance, and relieving the requirement for the energy expensive process of extruding drugs (Simões , 2003; Viegas et al., 2005). This model justifies the fact that drug efflux pumps appear to be required to a lower extent during exponential growth in the presence of stress, even when the stress agent is not degraded or neutralized. Indeed, no catabolism of clotrimazole or acetic acid, in the presence of glucose, is registered in yeast cells (Prasad et al., 2002; Guerreiro et al., 2012). Thus, when a new population is suddenly exposed to the vaginal environment or antifungal therapy or, more significantly, the two stresses at the same time, the expression of Aqr1 appears to be important to the success of colonization and persistence, but is not the single factor leading to chemical stress resistance.

This study, characterizing the *C. glabrata* Aqr1 multidrug transporter involved in flucytosine and azole drug resistance, highlights the importance of studying the remaining members of this family in *C. glabrata* in this context, with an expected impact in the treatment of the increasing number of drug resistant fungal infections. Significantly, CgAqr1 has close homologues in other pathogenic *Candida* species, e.g., orf19.9520 from *C. albicans*, CPAR2_501800 from *C. parapsilosis* or Cd36_51250 from *C. dubliniensis*, which may also play a role in acetic acid and antifungal drug resistance in these related pathogenic yeasts. So far, only the drug:H^+^ antiporters Mdr1 and Flu1, from *C. albicans* (Goldway et al., 1995; Calabrese et al., 2000), CgQdr2 from *C. glabrata* (Costa et al., 2013) and Mdr1 from *C. dubliniensis* (Sullivan et al., 2004) have been linked to antifungal drug resistance, more specifically to azoles. In *C. glabrata*, there are 15 predicted DHA transporters, of which 10 belong to the DHA1 family, predicted to have 12 transmembrane spanners, and 5 to the DHA2 family, predicted to have 14 transmembrane segments (Gbelska et al., 2006). The expectation that these transporters, that remain so far mostly uncharacterized, may play a significant role in multidrug resistance in *C. glabrata* is reinforced by the findings of this study.

## Conflict of Interest Statement

The authors declare that the research was conducted in the absence of any commercial or financial relationships that could be construed as a potential conflict of interest.
